# Post-infarct evolution of ventricular and myocardial function

**DOI:** 10.1007/s10237-023-01734-1

**Published:** 2023-07-05

**Authors:** K. L. P. M. Janssens, M. Kraamer, L. Barbarotta, P. H. M. Bovendeerd

**Affiliations:** Department of Biomedical Engineering, Eindhoven University of Technology, Eindhoven, 5600MB The Netherlands

**Keywords:** Computational modeling, Myocardial infarction, Ventricular remodeling, Cardiac patch, Infarct mechanics, Cardiac function

## Abstract

Adverse ventricular remodeling following acute myocardial infarction (MI) may induce ventricular dilation, fibrosis, and loss of global contractile function, possibly resulting in heart failure (HF). Understanding the relation between the time-dependent changes in material properties of the myocardium and the contractile function of the heart may further our understanding of the development of HF post-MI and guide the development of novel therapies. A finite element model of cardiac mechanics was used to model MI in a thick-walled truncated ellipsoidal geometry. Infarct core and border zone comprised 9.6 and 8.1% of the LV wall volume, respectively. Acute MI was modeled by inhibiting active stress generation. Chronic MI was modeled by the additional effect of infarct material stiffening, wall thinning and fiber reorientation. In acute MI, stroke work decreased by 25%. In the infarct core, fiber stress was reduced but fiber strain was increased, depending on the degree of infarct stiffening. Fiber work density was equal to zero. Healthy tissue adjacent to the infarct showed decreased work density depending on the degree of infarct stiffness and the orientation of the myofibers with respect to the infarct region. Thinning of the wall partially restored this loss in work density while the effects of fiber reorientation were minimal. We found that the relative loss in pump function in the infarcted heart exceeds the relative loss in healthy myocardial tissue due to impaired mechanical function in healthy tissue adjacent to the infarct. Infarct stiffening, wall thinning and fiber reorientation did not affect pump function but did affect the distribution of work density in tissue adjacent to the infarct.

## Introduction

Myocardial infarction (MI) is the irreversible death of cardiac muscle cells in part of the myocardium due to a prolonged lack of blood supply. Annually, an estimated 805.000 individuals are affected in the US alone with a mortality rate of 14% (Tsao et al. 2022). In patients that survive the acute phase of MI, deceased cardiomyocytes are replaced by fibrotic scar tissue over the course of several weeks. This tightly cross-linked, collagenous tissue has significant tensile strength to prevent rupture of the myocardial wall (Gupta et al. 1994; McGarvey et al. 2015; Fomovsky and Holmes 2010). However, pathological fibrosis and excessive deposition of collagen fibers within the scar tissue are important contributors to left ventricular (LV) dysfunction and play an important role in LV remodeling (Xie et al. 2013). Abnormal mechanical loading of the myocardium is thought to drive the remodeling process and promote infarct expansion and wall thinning. Overall, long-term adverse ventricular remodeling post-MI can induce LV dilation, diastolic dysfunction, ventricular tachycardia, and eventually lead to heart failure (HF) (French and Kramer 2007; Frantz et al. 2022).

Left ventricular assist devices (LVADs) are increasingly used in end-stage HF patients not eligible for transplantation. However, LVAD implantation as destination therapy does not constitute a lasting solution. Patients are often re-hospitalized in the first year post-implantation with high mortality and complication rates (Dunlay et al. 2016). These observations have sparked the development of alternatives to LVAD implantation. Cardiac patches, fixed to the epicardium, may provide appropriate mechanical support to the heart and stimulate remodeling pathways through cellular or molecular delivery. Some groups even aim to add functional support by incorporating active contractile tissue through tissue engineering techniques like the BRAVƎ project (https://projectbrave.eu/). Some devices have already been claimed to reduce infarct area, reverse ventricular remodeling and enhance ejection fraction and cardiac function (Li et al. 2022). Reverse remodeling is thought to be driven by the mechanical loading of the myocardium. So it is important to understand this local load, and its dependence on the mechanical properties of the patch and its interaction with the native cardiac tissue. In addition, it is important to understand the deficit in cardiac function caused by the infarction that should (partially) be restored by the cardiac patch. However, due to scar tissue maturation and LV remodeling both cardiac function and mechanical loading in tissue adjacent to the infarction vary over time, which may have implications for the mechanical support to be delivered by a cardiac patch (Richardson et al. 2015).

In this study, we aim to quantify the effect of the evolution of an acutely infarcted LV into a chronically remodeled state on global cardiac pump function and local myofiber mechanics. During this evolution, the LV is subject to a number of changes including infarct stiffening, wall thinning, reorientation of fibers, border zone expansion and LV dilation. This study places emphasis on the increase in tissue stiffness in the infarct region, but also considers local wall thinning and fiber reorientation, albeit to a lesser extent. We employ a finite element model of cardiac mechanics to assess global function by means of LV hemodynamics and local tissue function by analyzing stress and strain patterns.

## Methods

### Model of cardiac mechanics

**Fig. 1:**
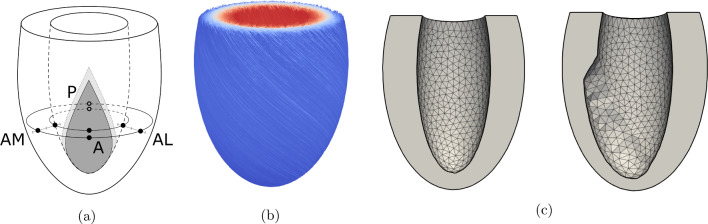
LV geometry with infarct region and border zone **a**, fiber orientation **b** and LV mesh **c** without (left) and with (right) wall thinning. For clarity, the contours of the transmural infarct are depicted on the endocardial surface only. Anterior sites are located in the infarct. Posterior (P), anterior-medial (AM) and anterior-lateral (AL) sites are located in the healthy tissue outside the border zone in both subepicardial and subendocardial layers. In (b), the helix angle $\alpha _h$ is plotted in colour

The finite element model of healthy cardiac mechanics from Bovendeerd et al. (2009) was used as a basis for this study. A brief description of this model is provided here. Section 2.2 explains modification to model acute and chronic MI. In its unstressed state, the geometry of the LV is approximated by a truncated thick-walled ellipsoid. Cavity and wall volumes were set to 44 and 136 ml, respectively (see Fig. 1a). The myocardium was modeled as a non-linearly elastic, transversely isotropic, nearly incompressible material with an active stress component that acts parallel to the direction of the myofibers (Eq. 1). The Cauchy stress tensor $\varvec{\sigma }$ is given by:

1 $$\begin{aligned} \varvec{\sigma } = \quad f_{\text {pas}} \varvec{\sigma }_{\text {pas}} \quad + \quad f_{\text {act}} \sigma _{act} \vec {e}_f \vec {e}_f \end{aligned}$$

Here $\vec {e}_f$ represents the unit vector in the fiber direction and $\varvec{\sigma }_{\text {pas}}$ and $\sigma _{\text {act}}$ represent the passive and active components of the total stress tensor, respectively. Both the active and passive stress models, including the parameters used, are identical to those presented in the 2009 study. Active stress was assumed to act along the fiber direction only. Multiplication factors $f_{\text {pas}}$ and $f_{\text {act}}$ were introduced to this equation to allow variation of material properties over the myocardium. They were set to a value of 1 to model the healthy LV (simulation REF), but could spatially deviate from 1 to model the infarction. A rule-based method was used to define the fiber orientation (see Fig. 1b) in terms of a helix-angle $\alpha _h$ and transverse angle $\alpha _t$. These angles represent the angle between the circumferential direction and the projection of the fiber vector on the circumferential-longitudinal and circumferential-transmural plane, respectively. Normalized Legendre polynomials were used to describe the nonlinear endo-to-epicardial and base-to-apex variation of $\alpha _h$ and $\alpha _t$ with parameter values according to simulation FIB in the 2009 study. The helix angle ranged from +70 degrees at the endocardial surface, through +20 degrees at the midwall until −50 degrees at the epicardial surface. The transverse angle was set to zero at the endocardial and epicardial surfaces. It varied approximately parabolically variation from the endocardial to the epicardial surface, with maxima of about −40 degrees and +10 degrees near the apex and base, respectively.

In the model, the equations for conservation of momentum were solved:

2 $$\begin{aligned} \vec {\nabla } \cdot \varvec{\sigma } = \vec {0} \end{aligned}$$

Rigid body motion was suppressed using Dirichlet boundary conditions. For all nodes in the basal plane, out-of-plane displacement was suppressed and in-plane displacement was confined by restricting the solution of this subset of nodes to its nullspace. Neumann boundary conditions were used to subject the endocardial surface to a uniform LV pressure $p_{\text {lv}}$. During the isovolumetric phases, $p_{\text {lv}}$ is determined by the mechanical equilibrium with the myocardial tissue at constant end-diastolic or end-systolic volume. During the filling and ejection phase, $p_{\text {lv}}$ is governed by a 0D closed-loop lumped parameter model of the systemic circulatory system. In this model, the aortic and mitral valves are modeled as ideal diodes. Arteries, veins and peripherals are represented through constant resistances and capacitances. A complete description of the 0D model, including used parameter values, can be found in Bovendeerd et al. (2009).

### Modelling myocardial infarction

A prolate ellipsoidal coordinate system was used to define the LV geometry. This coordinate system relates to the Cartesian system, with coordinates (x, y, z), according to:

3 $$\begin{aligned} x= &\,\, {} C \sinh (\xi ) \sin (\theta ) \cos (\phi )\nonumber \\ y= &\,\,{} C \sinh (\xi ) \sin (\theta ) \sin (\phi )\nonumber \\ z= &\,\,{} C \cosh (\xi ) \cos (\theta ) \end{aligned}$$

where $\xi$, $\theta$ and $\phi$ represent the radial, longitudinal and circumferential coordinate, respectively. In ellipsoidal coordinates the LV geometry is described by $\xi _{\text {endo}} \le \xi \le \xi _{\text {epi}}$, $\theta _{\text {base}} \le \theta \le \pi$ and $0 \le \phi \le 2\pi$ where $\xi _{\text {endo}} = 0.37$, $\xi _{epi} = 0.68$ and $\theta _{\text {base}} = \theta (z_{\text {base}}=24$ mm).

The region of infarcted tissue was modeled to be representative of an occlusion of the left anterior descending (LAD) artery. Its shape was based on experimental observations (Bovendeerd et al. 1996) and modeled as a triangular area within the longitudinal-circumferential plane, spanned by coordinates ($\theta$, $\phi$): ($\frac{\pi }{2}$, 0), ($\pi$, $0.6 \cdot \pi$) and ($\pi$, $-0.6 \cdot \pi$). This yields a droplet-shaped area spanning from equator to apex, roughly 4 cm wide at its widest point on the epicardium (see Fig. 1a). The transition from healthy to infarct tissue was modeled through a border zone with a width of 5 mm (Lee et al. 1981; Sakai et al. 1985). Within this border zone, factors $f_{pas}$ and $f_{act}$ varied linearly from healthy to infarct settings. The overall size of the infarct region, in- and excluding the border zone, comprises 17.7 and 9.6% of the total LV wall volume, respectively.

The effect of a change in material properties was investigated in the following models (see Table 1). Acute myocardial infarction is simulated in AMI where active stress generation was eliminated by setting $f_{\text {act}}$ to 0 within the infarcted area while leaving passive tissue stiffness unchanged by setting $f_{\text {pas}}$ to 1. Subsequently, infarct stiffening in chronic MI was simulated by increasing $f_{\text {pas}}$ 2-fold (CMI2), 5-fold (CMI5), 10-fold (CMI10), 20-fold (CMI20) and 100-fold (CMI100) within the infarct.

The effect of fiber orientation in the infarct region was investigated in simulation CMI5cf, in which CMI5 was extended by reorienting fibers in the infarct core in the circumferential direction, following observations from Clarke et al. (2016), Zhuan et al. (2019). In the border zone, the fiber direction was linearly interpolated from the healthy to infarct value based on the distance to the core infarct region.

Finally, we investigated the effect of wall thinning in simulation CMI5wt, in which simulation CMI5 was extended by reducing the wall thickness of the infarct region to 50% of its original value, based on the experimental data presented in McGarvey et al. (2015) (Fig. 1c). To this end, the epicardial surface was kept unchanged, but the endocardial surface was moved outward. Wall thickness varied linearly over the border zone from its infarct value to its normal value. Unloaded cavity volume and wall volume changed to 50.8 ml and 129.2 ml, respectively.

### Simulations and postprocessing

The model was implemented in the FEniCS open-source computing platform and benchmarked against the preceding SEPRAN implementation, used in Bovendeerd (2009) (chapter 2 of Barbarotta (2020)). The mesh contained 109608 degrees of freedom and 24202 quadratic tetrahedral elements yielding an average element size of 5.75 mm$^3$. Elements were assigned to either healthy myocardium, border zone or infarct tissue based on the position of their midpoint. A timestep of 2 ms was used. The model was solved using a 2.6 GHz 16-core Intel processor.

A total of 9 simulations were performed (see Table 1). For every simulation, 5 cardiac cycles were computed with a heart rate of 75 bpm such that a hemodynamic steady-state was achieved with a change in stroke volume of less than 1% between subsequent cycles. Data from only the last cardiac cycle was considered for analysis. Global cardiac function was assessed using the pressure-volume loops and stroke work, computed according to Eq. 4.

4 $$\begin{aligned} W = \int p \text {d}V \end{aligned}$$

Work density per unit of tissue volume was computed using Eq. 5.

5 $$\begin{aligned} w = \int \sigma _{\text {act}} \text {d}\varepsilon \quad \text {where} \quad \varepsilon = \ln \left( \frac{l_s}{l_{s,0}}\right) \end{aligned}$$

where $l_s$ and $l_{s,0}$ represent the actual and reference sarcomere length, respectively.

Furthermore, we performed a more detailed analysis of fiber stress and strain at eight sites in the cardiac wall. Four were placed underneath the epicardial surface at 8% of the total epi- to endocardial distance, with an identical longitudinal coordinate of $\theta = 0.6\pi$. Sites were located in the remote (P, $\phi =\pi$), infarct (A, $\phi =0$), apical-medial (AM, $\phi =-\frac{\pi }{4}$) and apical-lateral (AL, $\phi =\frac{\pi }{4}$) regions of the ventricle. The other four sites were placed at the same longitudinal and circumferential position near the endocardium, at 8% endo- to epicardial distance (see Fig. 1a). The helix angle $\alpha _h$ at this subepicardial and subendocardial level equals -45 and 64 degrees, respectively. Both fiber stress and strain over time were analysed as well as quantified into work density, maximum systolic stress and end-diastolic and end-systolic fiber strain.

## Results

### LV hemodynamics

**Fig. 2:**
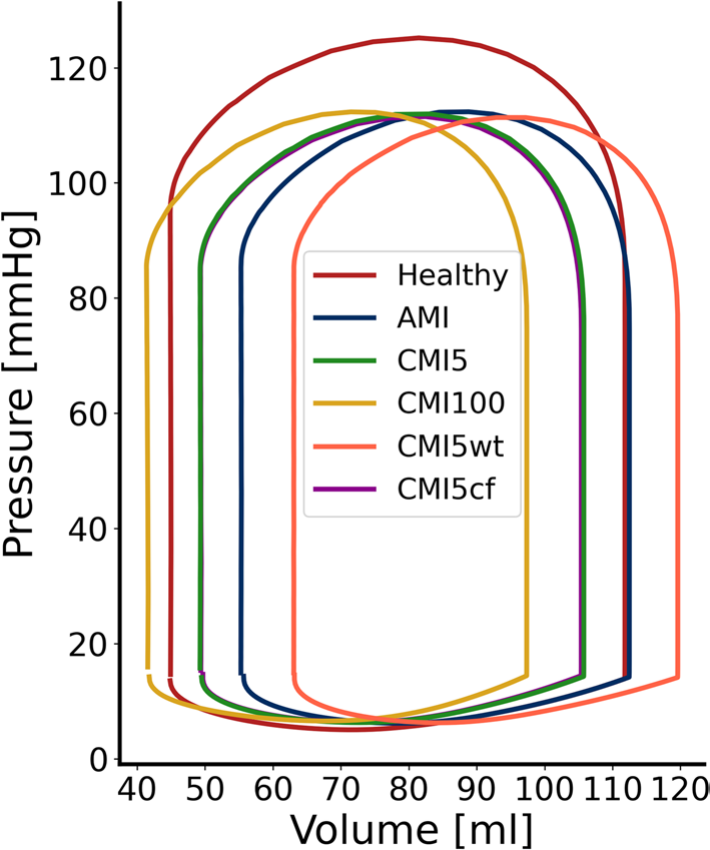
Pressure over volume plot of simulation REF (red), AMI (blue) and CMI5 (green), CMI100 (yellow), CMI5wt (orange) and CMI5cf (purple)

Figure 2 shows the LV pressure-volume loops for simulations REF, AMI, CMI5, CMI100,CMI5wt and CMI5cf. As compared to REF, infarct cases are characterized by a reduction of maximum LV pressure and stroke volume. With increasing infarct stiffness, the PV-loops shift to the left, but maximum LV pressure and stroke volume remain about constant. A quantitative hemodynamic summary for all 9 simulations is given in Table 1. The results found in REF are representative of the average adult with a maximum systolic pressure (p$_{\text {lv, max}}$) of 125 mmHg, cardiac output (CO) of 5.02 L/min and ejection fraction of 60%. Compared to REF, end-diastolic volume in AMI is preserved but the acute infarction leads to a 23% increase in end-systolic volume (ESV) corresponding to an approximate 15% reduction in stroke volume (SV), ejection fraction (EF) and cardiac output (CO). Combined with a lower p$_{lv, max}$, stroke work decreases by 24.5% in total. With increasing infarct stiffness, both ESV and EDV decrease by equal amounts with a maximum decrease of 14 ml in CMI100 when compared to simulation AMI. Hence, SV and CO remain relatively well-preserved and EF increases up to 57.5% in CMI100. Upon extending CMI5 with wall thinning in the infarct region (CMI5wt), both EDV and ESV increase by 14 ml compared to CMI5 which reduces EF to a value of 47.3%. Adding a change in fiber orientation to the circumferential direction in the infarct region (CMI5cf) induces no significant changes with respect to CMI5. Stroke work is similar in all simulations of MI.

**Table 1:** Summary of the definition of infarct properties through $f_{\text {act}}$ and $f_{\text {pas}}$, and hemodynamic results including end-diastolic volume (EDV), end-systolic volume (ESV), stroke volume (SV), ejection fraction (EF), cardiac output (CO), maximum systolic pressure ($p_{lv, max}$) and stroke work (SW)

Simulation	Infarct properties		Hemodynamic function						
	$f_{\text {act}}$ [−]	$f_{\text {pas}}$ [−]	EDV [ml]	ESV [ml]	SV [ml]	EF [%]	CO [l/min]	p$_{\text {lv, max}}$ [mmHg]	SW [J]
REF	1	1	112	44.9	67.0	59.8	5.02	125	0.98
AMI	0	1	112	55.3	57.2	50.9	4.27	112	0.74
CMI2	0	2	110	53	56.8	51.8	4.28	112	0.74
CMI5	0	5	106	49.3	56.5	53.4	4.24	112	0.73
CMI10	0	10	103	46.6	56.5	54.8	4.24	112	0.73
CMI20	0	20	101	44.4	56.7	56.1	4.25	112	0.74
CMI100	0	100	97.4	41.4	56.0	57.5	4.21	112	0.72
CMI5wt	0	5	120	63.1	56.5	47.3	4.24	111	0.73
CMI5cf	0	5	105	49.3	56.1	53.2	4.21	112	0.72

### Local mechanics

**Fig. 3:**
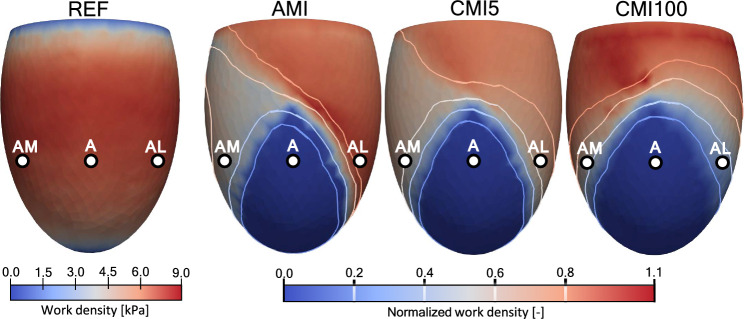
Work generated by fibers over the cardiac cycle, according to equation 5, for simulation REF. The figures for simulations AMI, CMI5 and CMI100 show normalized work with respect to the healthy simulation

Figure 3 shows work density in the anterior view of the LV as generated by myofibers over the entire cardiac cycle for simulations REF, AMI, CMI5 and CMI100. In REF, the spatial distribution is rotationally symmetric, with an average value of 7.3 ± 1.8 kPa. Towards the basal plane and apex, work density decreases, approaching a value of 0. To evaluate changes in work density for AMI, CMI5 and CMI100, the right 3 panels in figure 3 show work density $\bar{w}$, normalized to that in REF. In all cases of MI, $\bar{w}$ equals 0 within the infarct region and about 1 in the remote region. The border zone and healthy tissue adjacent to the border zone show large differences between simulations. In acute MI, $\bar{w}$ is reduced to 50% near site AM but increased to 110% near AL. When infarct stiffness increases 5-fold in CMI5, the amount of generated work near site AM recovers to 65% but drops to 70% near AL. This trend continues with further stiffening of the infarct in CMI100 where $\bar{w}$ decreases towards 30% in AL and increases up to 100% near AM. In AMI, CMI5 and CMI100 values of work density near the base are close to zero, just as in REF. The near-zero basal values of work density in REF cause normalized values $\bar{w}$ near the base to fluctuate strongly. Therefore, basal values have been set to 1.0 to improve visibility.

**Fig. 4:**
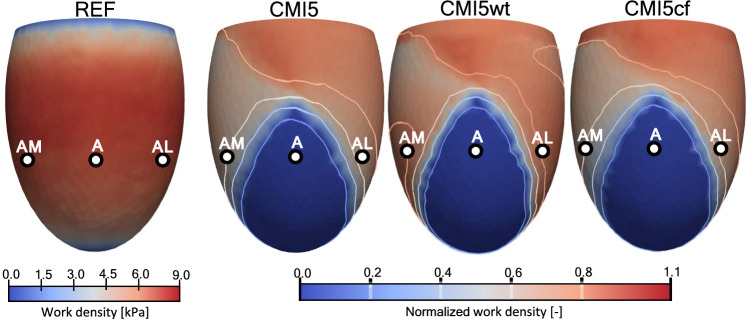
Work generated by fibers over the cardiac cycle, according to equation 5, for simulation REF. The figures for simulations CMI5, CMI5wt and CMI5cf show normalized work with respect to the healthy simulation

Figure 4 shows the effect when CMI5 is extended with wall thinning (CMI5wt). Normalized work density $\bar{w}$ increases from 65 to 80% at site AM, and from 70 to 80% at site AL. With a circumferential orientation of fibers in the infarct region (CMI5cf), $\bar{w}$ decreases from 65 to 53% at site AM, and increases from 70 to 72% at site AL.

**Fig. 5:**
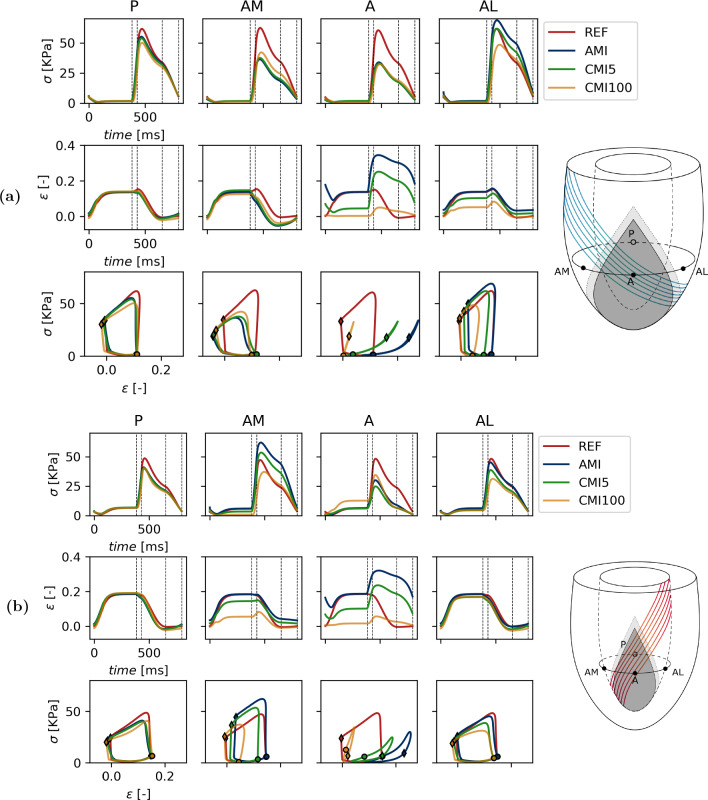
Local myofiber mechanics (rows) in 4 subepicardial (**a**) and subendocardial (**b**) points (columns). From the top to bottom row, Cauchy stress in fiber direction and logarithmic strain as a function of time and stress–strain loops for a healthy heart, AMI, CMI5 and CMI100. The dotted lines mark the diastolic, isovolumic contraction, systolic and isovolumic relaxation phase, respectively. End-diastole and end-systole are marked with a $\circ$ and $\lozenge$ respectively

**Fig. 6:**
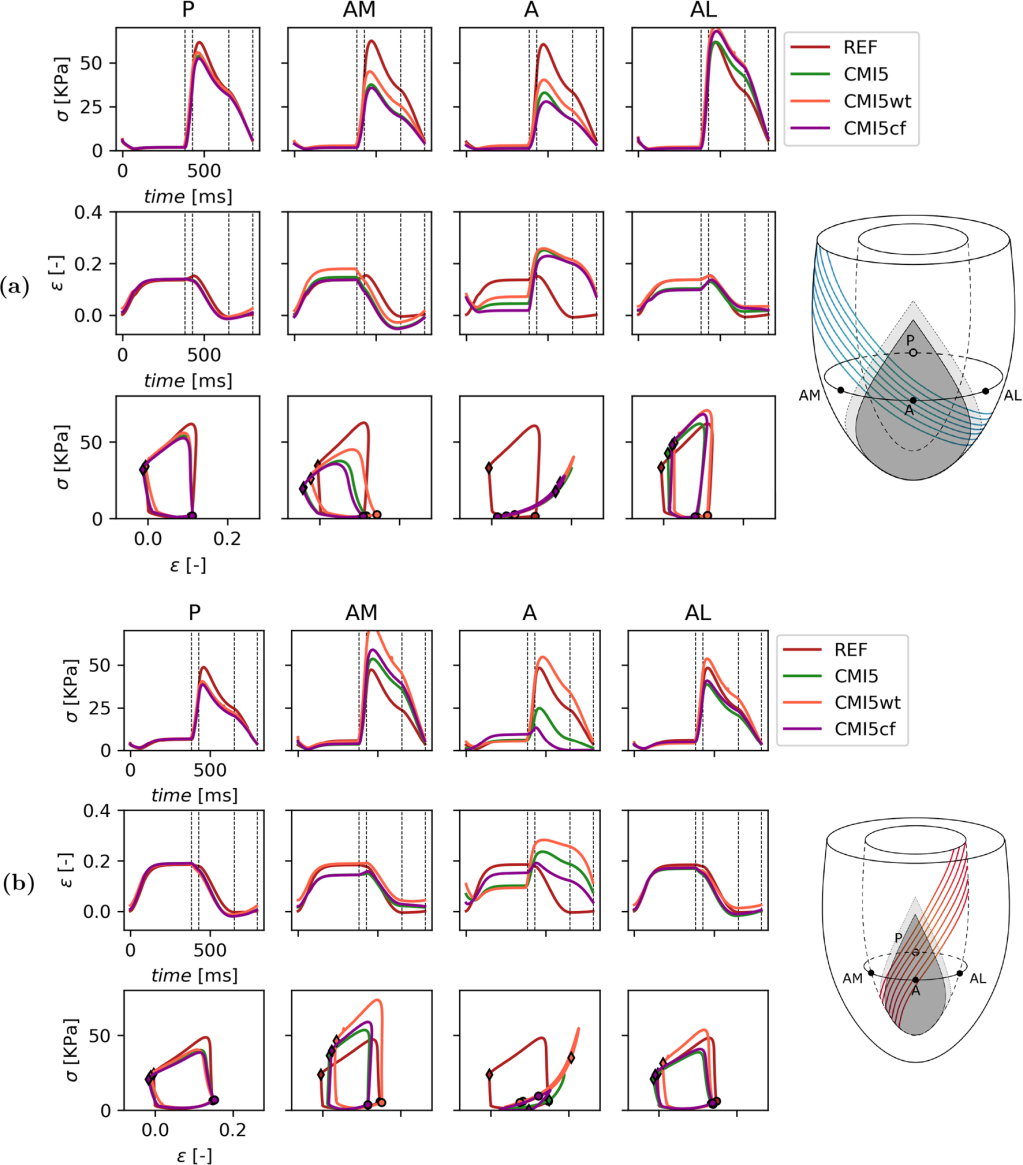
Local myofiber mechanics (rows) in 4 subepicardial (**a**) and subendocardial (**b**) points (columns). From the top to bottom row, Cauchy stress in fiber direction and logarithmic strain as a function of time and stress–strain loops for a healthy heart, CMI5, CMI5wt and CMI5cf. The dotted lines mark the diastolic, isovolumic contraction, systolic and isovolumic relaxation phase, respectively. End-diastole and end-systole are marked with a $\circ$ and $\lozenge$ respectively

**Fig. 7:**
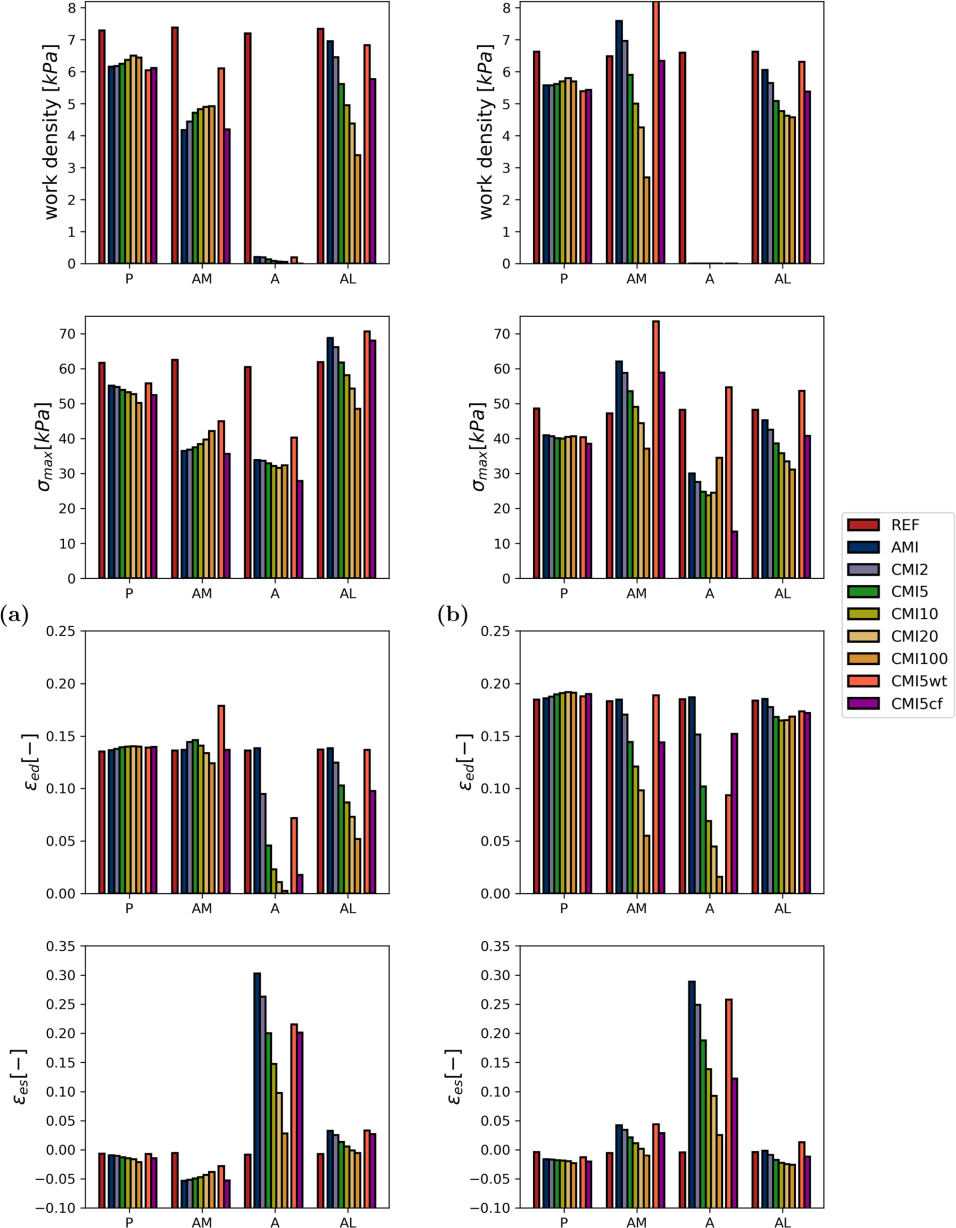
Overview of local metrics as measured subepicardially (**a**) and subendocardially (**b**) for the healthy heart, AMI, CMI2, CMI5, CMI10, CMI20 and CMI100. From top to bottom: work density, maximal Cauchy stress, end-diastolic sarcomere length and end-systolic sarcomere length

A more detailed view of local tissue mechanics is presented in Fig. 5 for simulations REF, AMI, CMI5, CMI100 and in Fig. 6 for simulations REF, CMI5, CMI5wt and CMI5cf. A more detailed view of local tissue mechanics for simulations REF, AMI, CMI5 and CMI100 is found in Fig. 5. Here, fiber stress and strain are shown as a function of time and fiber stress is plotted vs. fiber strain in the four subepicardial points indicated in Fig. 1a. Work density, maximum fiber stress, end-diastolic fiber strain and end-systolic fiber strain are quantified in Fig. 7 for all simulations.

We first consider the subepicardial level (Figs. 5a and 7a). In the healthy heart (REF), local tissue mechanics are identical in all four sites due to rotational symmetry (Fig. 5). Fiber stress and strain reach a plateau at end-diastole of 1.6 kPa and 0.135, respectively. During isovolumic contraction and early ejection, fiber stress increases towards 61 kPa and fiber strain increases slightly by 0.02. During the ejection and isovolumic relaxation phase, both fiber stress and fiber strain gradually drop to 0.8 kPa and 0.01, respectively. Work density, which corresponds to the area enclosed by the stress–strain loop, equals 7.3 kPa.

At the posterior site P, all simulations of MI show behaviour similar to that in REF. Maximum stress decreases by about 10% in AMI. It decreases further with increasing infarct stiffness, by about 20% in CMI100. The slight increase in fiber strain during isovolumic contraction disappears though end-diastolic and end-systolic strain remains relatively constant irrespective of the degree of MI. Work density decreases by about 15% in AMI though infarct stiffening restores up to 5% of this loss in CMI20 (Fig. 7).

Within the infarcted tissue (site A), peak fiber stress decreases by roughly 45% in all cases of MI. This stress is passive since $f_{\text {act}}$ = 0. In AMI, fiber strain over the course of diastole is identical to that in REF. Subsequently, fibers stretch further during isovolumic contraction compared to REF to a strain of about 0.35. This elevated strain is sustained through the ejection and part of the isovolumic contraction phase. The surface area enclosed by the stress–strain loops disappears completely and 0 kPa in work density is found. With increasing stiffness of the infarct, end-diastolic fiber strain decreases towards its unloaded reference of 0 in CMI100. Compared to AMI, the maximum fiber strain that is reached during contraction decreases with higher infarct stiffness. In CMI5 and CMI100 a value of 0.26 and 0.05 is reached, respectively. The stress–strain loops steepen with increasing infarct stiffness and remain closed.

Site AM shows a decrease in peak fiber stress of over 24 kPa in AMI, as compared to REF. End-diastolic fiber strain remains unchanged but fibers are found to shorten early during isovolumic contraction. Furthermore, fiber strain is reduced by about 0.1 during the entire ejection phase. Due to these changes, the stress–strain loop skews to the left and work density is reduced by 3.1 kPa compared to REF. Peak fiber stress increases with infarct stiffness and up to 7.1 kPa can be restored in CMI100 as compared to AMI. End-diastolic fiber strain remains relatively unaffected though a slight increase of 0.02 is found in CMI2 and CMI5. Any further stiffening results in a reduction of this length. Early fiber shortening decreases with higher infarct stiffness and causes stress–strain loops to become less skewed. Consequently, work density increases and converges to a value of 5.4 kPa in CMI100.

Site AL shows increased peak fiber stress of 70 kPa in AMI as compared to REF. Fiber strain is similar to that in REF over the duration of the entire cardiac cycle. Only the amount of shortening decreases by 0.03 and consequently, end-systolic sarcomere length is higher. Compared to REF, the stress–strain loop in AMI is higher and narrower and only a minor reduction in work density of 0.4 kPa is found. Higher infarct stiffness leads to a reduction in peak fiber stress when compared to AMI, with values of 61 and 48 kPa in CMI5 and CMI100, respectively. End-diastolic fiber strain drops with infarct stiffness to 0.05 in CMI100 while end-systolic fiber strain is similar to that in REF. Stress–strain loops shift to the left and decrease in size depending on the degree of infarct stiffening. A third of work density is lost in CMI10 and over 50% in CMI100 (Fig. 7a).

Additionally, local tissue mechanics were analysed at subendocardial level and are shown in Figs. 5b and 7b. Some of the trends found subepicardially are also observed here. In the remote region P, the effect of infarction is minor and comparable to that at the subepicardium. Also in the core of the infarct (site A), the effect of infarct stiffening is similar at the subendocardium and subepicardium. In contrast to the behaviour observed epicardially, stress–strain loops are not closed and traversed in a clockwise direction. While this suggests that energy is dissipated here, this is not the case, as will be explained in the discussion. The results obtained at the subendocardial AM site do not resemble those at the subepicardial AM site. Instead, they are similar to those at the subepicardial AL site. This symmetry is not found when comparing the subendocardial AL site with the subepicardial AM site. In fact, the effect of acute and chronic infarction at this site is more comparable to that in the remote subendocardial P site.

Figure 6 shows the local mechanics for simulations REF, CMI5, CMI5wt and CMI5cf. Again we first consider the subepicardial level (Figs. 6a and 7a). When CMI5 is extended with wall thinning (CMI5wt), small changes occur in both peak fiber stress and end-systolic fiber strain near site P which lead to a small decrease in work density of 0.3 kPa. Within the infarct site (A), thinning of the ventricular wall leads to increased end-diastolic fiber stress and strain from 1.6 and 0.04 to 2.8 kPa and 0.07, respectively. The elevated stress is maintained throughout the ejection phase though fiber strain follows an almost identical course to CMI5. In site AM, CMI5wt shows an increase in peak fiber stress from 37 kPa to 45 kPa compared to CMI5. End-diastolic and end-systolic fiber strain are also elevated by 0.05 and 0.04, respectively, which leads to an increase in work density of 1.4 kPa. In site AL, peak fiber stress increases from 61 to 70 kPa and end-diastolic and end-systolic fiber strain increase by 0.03 compared to CMI5 causing work density to increase to 6.8 kPa. With a change of fiber orientation in the circumferential direction in the infarct region (CMI5cf), only small changes are observed near the remote site P. Site A shows reduced end-diastolic fiber stress and strain from 1.6 and 0.04 to 1 kPa and 0.1, respectively. Peak fiber stress is reduced by 6 kPa though fiber strain largely follows the same course as in CMI5. In site AM, only a small decrease in fiber strain is found and the stress–strain loop is skewed slightly further compared to CMI5. In site AL, an increase in peak fiber stress is found while fiber strain remains almost identical to CMI5 throughout the cardiac cycle. Work density increases slightly by 0.2 kPa.

In the subendocardial region of the ventricle (Figs. 6b and 7b), little changes are found in the posterior site P. When extending CMI5 with wall thinning, fiber stress and strain increase in the infarct site (A). In sites AM and AL end-diastolic fiber strain and peak fiber stress is increased compared to CMI5. Subsequently, work density also increases from 5.9 and 5.0 kPa to 8.0 and 6.3 kPa for AM and AL, respectively. With a circumferential orientation of fibers in the infarct region, the only notable differences are found in the infarct region (A). Here, end-diastolic fiber strain is increased whilst both fiber stress and strain during the ejection phase are decreased.

## Discussion

In this study, we assessed the effects of infarct stiffening on global cardiac function and local myofiber mechanics during the transition from an acutely infarcted towards a chronically infarcted state. In addition, we investigated the effect of thinning and fiber reorientation in the infarct region.

### Cardiac pump function in MI

An acute infarct with a core and boundary region comprising 9.6 and 8.1% of the LV wall, respectively, was found to cause a 24–26% loss of pump function (Fig. 2, Table 1). Stiffening of the infarct causes a leftward shift of the pressure-volume loop, whilst stroke volume remains constant. This disproportional loss in pump function can be attributed to a loss in tissue function that extends outside the infarct and border zone into the area with normal passive and contractile properties (see Fig. 3). The extent of this loss depends on the location in the LV wall and the degree of infarct stiffening.

Overall, pump function decreases by 26% after AMI but is hardly affected by subsequent stiffening of the infarct region. Our results show that this stiffening does affect local tissue function, with a partial recovery in fibers arranged in series with the infarct and a further decline in function in fibers arranged in parallel to the infarct. These effects seem to offset each other evenly as pump function varies no more than 2% between simulations of MI. Thinning of the ventricular wall or circumferential orientation of collagen fibers in the infarct region induced minor changes in pump function. The pressure-volume loop shifted rightward with reduced wall thickness but stroke volume and work are identical to CMI5. This rightward shift is partially caused by the increase in cavity volume that arises from the fact that we modeled infarct thinning by an outward displacement of the endocardial surface.

### Myofiber function in MI

When the infarct is modeled exclusively through a change in material properties, only minor differences are observed in healthy tissue distant from the infarct when comparing healthy and infarct cases (Figs. 5 and 7, column P). The main effect is a slight decrease in fiber stress, related to the slight decrease in LV pressure. The remote region is situated at such a distance that any mechanical changes induced at the infarct site have faded. Within the infarct tissue (site A), the lack of any contractile ability leaves tissue that solely relies on its passive material properties. The stress needed to achieve mechanical equilibrium and counteract active stress from the healthy tissue and LV cavity pressure can only be achieved by stretching of the tissue. This stress is lower than the stress in the healthy case, but similar is identical for all three infarct cases. The fiber strain to achieve this stress reduces as infarct stiffness increases. This reduction of fiber strain causes a reduction of diastolic filling and leads to a lower end-diastolic volume as observed in CMI5 and CMI100 (see Fig. 2). It also leads to a reduction of the outward bulging of the tissue during ejection.

In the healthy tissue adjacent to the infarct myofiber function depends on location. In the medial subepicardial region (site AM) work density decreases by about 45% in AMI (see Figs. 5a and 7a). This functional deterioration can be related to the local arrangement of fibers with respect to the infarction. The subepicardial helix angle of -45 degrees places tissue near AM in a series arrangement with the infarct. Thus, in acute MI, stress generated by these fibers is exerted onto relatively compliant infarct tissue, leading to early shortening, less stress generation and decreased work density over a cardiac cycle. Infarct stiffening counteracts this effect so that part of the lost work density is recovered. In the lateral subepicardial region (site AL) fibers are placed in a more or less parallel arrangement with the infarct. In AMI, these lateral fibers shorten less during ejection as they are tethered to the stretching infarct region. Being longer, they can also generate more active stress via the Frank-Starling mechanism. As the infarct stiffens, stretching of the infarct during diastole is reduced. Consequently, stretching of the AL fibers and stress generation is reduced as well. Shortening of these fibers during ejection is also hampered by the presence of a stiff infarct. This leads to an increasing reduction in work density as the infarct stiffens (Fig. 7a).

The mechanisms by which fiber function may be impaired are not limited to the subepicardium alone. At the subendocardial level, similar trends are observed when taking into account the local fiber orientation. Due to the change in the sign of the helix angle, regions arranged in series and parallel with the infarct are switched. Consequently, epicardial behaviour observed lateral to the infarct, where fibers are in parallel to the infarct region, is found medially in endocardial tissue. However, the effect of serial arrangement of the fibers, which was clearly visible at the epicardial AM site, is not obvious at the endocardial AL site, suggesting that more effects play a role here. In general, changes in stress and strain time courses result from complex 3D interactions with the surrounding tissue, in which the serial and parallel effects act together to a different degree.

**Fig. 8:**
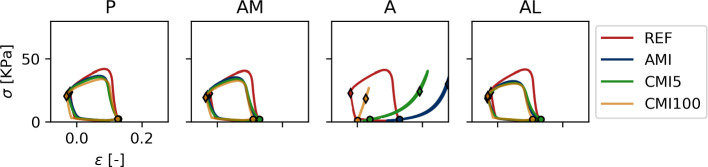
Stress–strain loops in 4 points at 60% of the total endo to epicardial distance. At this transmural level, the helix angle equals 0

To further evaluate the role of fiber orientation with respect to the infarct region, we analyzed local mechanics at the four sites at a transmural distance of 60% from endo- to epicardium (see Fig. 8). At this position, the helix angle equals 0 and fibers are oriented circumferentially, in series arrangement with the infarct region. Indeed, at this transmural level the stress–strain loops at sites AM, AL and P are similar and resemble those in serial subepicardial site AM and subendocardial site AL (see Fig. 5).

Thinning of the infarct site (CMI5wt) leads to more bulging of the infarcted tissue that is reflected in an increase of fiber end diastolic and end systolic fiber strain, both in the infarct (site A) and in sites adjacent to the border zone (AM and AL), see Fig. 6. Via the Frank-Starling effect, this elevated strain leads to an increase in active stress and work density at sites AL and AM. Thus wall thinning seems to mitigate the primary detrimental effect of the infarct in the neighbouring healthy tissue, as resulting from a change in material properties.

The effect of adding a circumferential fiber orientation on top of the 5-fold infarct stiffening (CMI5cf vs CMI5) on mechanics in sites AL and AM is similar to the effect of reducing the infarct stiffness (CMI2 vs CMI5). This can be explained from the fact that fibers at sites AM and AL are not in parallel to the direction of largest stiffness in the infarct anymore. Overall, the differences with respect to CMI5 are relatively small and it appears that the change in passive tissue stiffness plays a more prominent role.

In all infarct cases, the area enclosed in the stress–strain loops in the infarct at the subendocardial site A is non-zero despite the absence of contractility. Closer inspection reveals higher stress during the first half of the cardiac cycle compared to the second, as denoted by the $\lozenge$ and $\circ$ respectively. This suggests that energy is dissipated here, even though the tissue is modeled to behave elastically. However, it must be noted that the loading state of the tissue is not purely uniaxial: to fully evaluate work density, the double dot product of the full 3D stress and strain tensor must be evaluated. Such evaluation shows that no energy is created or dissipated here.

### Study limitations

In the design of this study we have chosen to model the infarct through an absence of active stress development and an increase in passive tissue stiffness. We assumed a simple model for the border zone, with a linear variation of active and passive tissue properties (through $f_{\text {act}}$ and $f_{\text {pas}}$) over a region with constant width. Thus we did not account for the finding that the actual variation of tissue properties over the border zone may be non-linear, and that the width of the border zone may change in time (Shimkunas et al. 2014; Jackson et al. 2002). We also considered a reduction in wall thickness and a change in fiber orientation within the infarct region. The changes in passive tissue stiffness and wall thickness cover the ranges reported in literature (Gupta et al. 1994; McGarvey et al. 2015; Fomovsky and Holmes 2010; Hiesinger et al. 2012; Richardson et al. 2015) and a more circumferential orientation of collagen fibers in infarct tissue has been previously reported (Clarke et al. 2016; Zhuan et al. 2019). However, ventricular growth and remodelling can also induce geometric changes to the ventricle that have not been taken into account in this study. LV-dilation post-MI may increase left ventricular volume, shifting the pressure-volume loop to the right. Infarct expansion may increase infarct size and lead to a further decrease in cardiac pump function. Such changes can be readily implemented in our model, but we choose to limit the number of variations in the current study to keep the analysis tractable.

A rotationally symmetric, ellipsoidal geometry and fiber field were used in our model. As compared to using a more realistic patient-specific geometry, this approach allows for a better evaluation of the effect of an infarct on fiber mechanics: since sites P, A, AM and AL show identical mechanics in the healthy state, differences between sites in infarct conditions can be exclusively attributed to the presence of the infarct.

We also assumed simultaneous activation of myofibers and did not take the right ventricle into account. It has been suggested that electrophysiological factors play a minor role in changes in mechanical function upon infarction (Leong et al. 2017). While there are no fundamental difficulties in including a realistic biventricular geometry and asynchronous activation in our model, the interpretation of the results of such a model will become increasingly difficult. Despite these simplifications, we expect the mechanism of series and parallel coupling to remain an important determinant of local tissue mechanics.

Finally, we did not account for regulatory mechanisms that occur in response to an infarct and might increase both peripheral resistance and the amount of blood in the circulatory system Richardson et al. (2015). This allows for the comparison of simulations purely based on the effect of MI but also complicates a direct comparison to patient data. Capturing all mechanisms that affect LV hemodynamics post-MI within the model may be technically feasible but was considered outside the scope of this study. Furthermore, whilst regulatory mechanisms might restore cardiac pump function in terms of cardiac output and mean arterial pressure they would not change the spatial inhomogeneities in tissue function, observed in our study.

### Literature comparison

Several numerical studies have investigated the effect of an infarct on global cardiac function and local myofiber mechanics. Janz and Waldron (1978) developed a finite element model of the infarcted heart at diastole, assuming isotropic material properties. From their results, they hypothesized that muscle fibers in close proximity to a chronic infarct are restrained in end-diastolic length resulting in a less optimal position on the Frank-Starling curve. Bogen et al. (1980) presented an isotropic membrane model of the infarcted LV during diastole and systole. They concluded that the mechanical disadvantage of the infarct includes mechanically induced contraction derangement in the non-infarcted myocardium. To our knowledge, these models showed the earliest evidence for mechanical tethering of healthy to infarct tissue even though they assumed highly simplified cardiac mechanics. Furthermore, both Janz and Bogen found that increased infarct stiffness reduces bulging of the infarction and improves systolic function at the expense of diastolic compliance of the ventricle, in agreement with our study.

Bovendeerd et al. (1996) presented a model of the LV with an acute infarct, assuming anisotropic passive and active material properties. They introduced the concept of the serial and parallel border zone to explain their results. In this paper, we extend their analysis by investigating the effect of long-term stiffening of the infarct. In addition, we replaced their open-loop circulatory model by a closed-loop model to be able to better capture hemodynamic effects such as changes in end-diastolic filling pressure and ejection pressure. We also increased the mesh resolution allowing us to define the outline of the infarcted region and border zone more precisely and solve the model more accurately.

Mazhari et al. (2000) used an anatomically realistic finite element model of the dog heart to assess differences in epicardial end-systolic strain between a control case and acute LAD and LCx infarcts. A functional border zone was defined as the region with higher end-systolic remodeling strain when compared to control. A wider functional border zone was found in the cross-fiber direction compared to the fiber direction, caused by increased wall stress arising from mechanical interaction between ischemic and non-ischemic myocardium (Mazhari et al. 2000). We did not focus on the size of dysfunctional tissue but Fig. 5a does show higher end-systolic fiber strain in parallel regions while it is decreased in serial regions which agrees with behaviour found in the cross-fiber and fiber direction, respectively.

Guccione et al. (2001) modeled the diastolic and isovolumic contraction phase in the sheep heart with a 10% infarct region and 14% border zone, 10 weeks after infarct induction. Their findings on midwall fiber strain in both the acute and chronic infarct are similar to our study. Moreover, only small differences in midwall fiber strain were observed between posterior and anterior regions of the border zone and these differences decreased with higher stiffness of the infarct. However, the significant amount of fiber shortening during isovolumic contraction observed by Guccione in the remote region of the ventricle is not in agreement with our study. Since the series and parallel effects outlined in this paper are sensitive to geometric effects, these differences may be explained by the use of a patient-specific geometry and the specific location where stress and strain are evaluated.

Walker et al. (2005) performed a similar analysis of the sheep heart with a 25% infarcted region at 22 weeks after infarct induction. They found a 24% increase in midwall end-systolic fiber stress in the border zone as compared to remote regions. Our study suggests that changes in stress vary as a function of location in the border zone and the corresponding orientation of fibers.

Remme and Smiseth (2007) simulated a reduced rate and magnitude of active fiber stress development in the border zone of an infarcted LV. The remote region was found to stretch the border zone during early systole to maintain mechanical equilibrium. Our results indicate that this behaviour may only be found in fibers that are arranged in series with the infarction but is less apparent in parallel fibers.

Fomovsky et al. (2011) found that isotropic stiffening of the infarct reduced end-diastolic (EDV) and end-systolic volume (ESV) volume but had little effect on stroke volume. End-diastolic strain in the healthy heart and acute MI was identical and uniform systolic contraction was converted to passive stretching of the infarct during ischemia. Isotropic stiffening reduced both end-diastolic and end-systolic stretching of the infarct, which is in agreement with our study. However, they found large variations in end-systolic stress within the infarct after acute and chronic MI whereas these remained relatively constant in our study.

Leong et al. (2017) used a rotationally symmetric ellipsoidal finite element model of cardiac mechanics to assess the effect of infarct transmurality on local fiber mechanics. With a full transmural infarct, 14.3% in size with a 3-fold increase in passive stiffness, the drop in SV and $p_{\text {lv, max}}$ was similar to those found in our simulations CMI2 and CMI5, in agreement with our result that cardiac function decreased disproportionally to infarct size as well. Furthermore, end-systolic fiber stress in the border and adjacent remote zones was higher when fibers were oriented tangential to the infarct boundary while it was suppressed in a perpendicular configuration. This trend corresponds with the parallel and series effects found in our results. However, they found fiber stress to be highest at end ejection, whereas our study suggests that it is highest at begin ejection. Consequently, the shape of the stress–strain loops is different as well. In addition, infarct-induced changes in maximum stress were much larger than those in our study. Similar discrepancies are observed when comparing our results with those of a more recent study by this group, that employs patient-specific geometries (Leong et al. 2021). Possibly these discrepancies are related to the choice of the fiber orientation field.

### Clinical implications

The present study shows how unfavourable mechanical loading conditions in the border zone and adjacent healthy myocardium lead to a relative loss of global cardiac function that exceeds the relative loss in healthy tissue. This pathological aspect of local fiber mechanics may be important in understanding the progression of MI into HF and the development of treatment to prevent adverse remodeling. Changes in mechanical load have been suggested to trigger a maladaptive growth and remodeling (G &R) response that may eventually lead to HF (Boulet and Mandeep 2021; Sutton and Sharpe 2000; Tsuda 2021; Schwinger et al. 1994; Weber et al. 1987; Ferrari et al. 2016). Our finding that the change in mechanical load varies in character and location as the infarct stiffens, might be important to better understand the G &R response in this region.

Cardiac patches may be used to limit the loss of pump function and the progression into HF. Passive patches can limit the change in mechanical load in this healthy tissue, either by direct mechanical interaction with this tissue or by indirect interaction through reduction of systolic bulging of the acutely infarcted tissue (Yao et al. 2022). To understand these interactions, the concept of serial and parallel muscle fibers might be helpful. Active, contractile patches have the potential to further restore cardiac function. To fully restore cardiac function, and compensate for the disproportional loss caused by the infarct, our study suggests that the amount of tissue within the patch should exceed the amount of native tissue lost. Furthermore, the mechanical interaction between the cardiac patch, infarct tissue and healthy myocardium may be important to ensure functionality of the patch. Dedicated research is required to outline an optimal design and point-out key design parameters that are important determinants of patch efficiency.

## Conclusion

This study shows that the relative loss in pump function in the infarcted heart exceeds the relative loss in healthy myocardial tissue. This disproportional loss in pump function is attributed to the impairment of mechanical function of fibers in healthy tissue adjacent to the infarct, because of an unfavourable mechanical interaction with the infarct tissue. As passive tissue stiffness in the infarct evolves from an acute state to a chronic state, the spatial distribution of the impaired tissue varies, but the overall effect on pump function remains about the same. Contractile function in the healthy tissue adjacent to the infarct was increased as a consequence of thinning of the infarct region. It was affected by the reorientation of fibers in the infarct region to a much lesser extent. These findings may be important for a better understanding of post-infarct growth and remodeling and guide the design of biological ventricular assist devices.

## Data Availability

The data that support the findings of this study are available from the corresponding author upon reasonable request.

## References

[CR1] Barbarotta L (2020). Towards computer assisted cardiac medicine: sensitivity analysis and data assimilation in models of left ventricular mechanics.

[CR2] Bogen DK, Rabinowitz SA, Needleman A (1980). An analysis of the mechanical disadvantage of myocardial infarction in the canine left ventricle. Circ Res.

[CR3] Boulet J, Mandeep RM (2021). Left ventricular reverse remodeling in heart failure: remission to recovery. Structural Heart.

[CR4] Bovendeerd PHM, Arts T, Delhaas T (1996). Regional wall mechanics in the ischemic left ventricle: numerical modeling and dog experiments. Am J Physiol Heart Circ Physiol.

[CR5] Bovendeerd PHM, Kroon W, Delhaas T (2009). Determinants of left ventricular shear strain. Am J Physiol Heart Circ Physiol.

[CR6] Clarke SA, Richardson WJ, Holmes JW (2016). Modifying the mechanics of healing infarcts: is better the enemy of good?. J Mol Cell Cardiol.

[CR7] Dunlay SM, Strand JJ, Wordingham SE (2016). Dying with a left ventricular assist device as destination therapy. Circ Heart Failure.

[CR8] Ferrari R, Malagù M, Biscaglia S (2016). Remodelling after an infarct: crosstalk between life and death. Cardiology.

[CR9] Fomovsky GM, Holmes JW (2010). Evolution of scar structure, mechanics, and ventricular function aftermyocardial infarction in the rat. Am J Physiol Heart Circ Physiol.

[CR10] Fomovsky GM, Macadangdang JR, Ailawadi G (2011). Model-based design of mechanical therapies for myocardial infarction. J Cardiovasc Transl Res.

[CR11] Frantz S, Hundertmark MJ, Schulz-Menger J (2022). Left ventricular remodelling post-myocardial infarction: pathophysiology, imaging, and novel therapies. Eur Heart J.

[CR12] French BA, Kramer CM (2007). Mechanisms of post-infarct left ventricular remodeling. Drug Discovery Today Disease Mech.

[CR13] Guccione JM, Moonly SM, Moustakidis P (2001). Mechanism underlying mechanical dysfunction in the border zone of left ventricular aneurysm: a finite element model study. Ann Thorac Surg.

[CR14] Gupta KB, Ratcliffe MB, Fallert MA (1994). Changes in passive mechanical stiffness of myocardial tissue with aneurysm formation. Circulation.

[CR15] Hiesinger W, Brukman MJ, McCormick RC (2012). Myocardial tissue elastic properties determined by atomic force microscopy after stromal cell-derived factor 1$$\alpha $$ angiogenic therapy for acute myocardial infarction in a murine model. J Thorac Cardiovasc Surg.

[CR16] Jackson B , Gorman J, Moainie S (2002). Extension of borderzone myocardium in postinfarction dilated cardiomyopathy. J Am College Cardiol.

[CR17] Janz RF, Waldron RJ (1978). Predicted effect of chronic apical aneurysms on the passive stiffness of the human left ventricle. Circ Res.

[CR18] Lee J, Ideker R, Reimer K (1981). Myocardial infarct size and location in relation to the coronary vascular bed at risk in man. Circulation.

[CR19] Leong CN, Lim E, Andriyana A (2017). The role of infarct transmural extent in infarct extension: a computational study. Int J Numer Methods Biomed Eng.

[CR20] Leong CO, Leong CN, Liew YM (2021). The role of regional myocardial topography post-myocardial infarction on infarct extension. Int J Numer Methods Biomed Eng.

[CR21] Li M, Wu H, Yuan Y (2022). Recent fabrications and applications of cardiac patch in myocardial infarction treatment. View.

[CR22] Mazhari R, Omens JH, Covell JW (2000). Structural basis of regional dysfunction in acutely ischemic myocardium. Cardiovasc Res.

[CR23] McGarvey JR, Mojsejenko D, Dorsey SM (2015). Temporal changes in infarct material properties: an in vivo assessment using magnetic resonance imaging and finite element simulations. Ann Thoracic Surg.

[CR24] Remme EW, Smiseth OA, Sachse FB, Seemann G (2007). Characteristic strain pattern of moderately ischemic myocardium investigated in a finite element simulation model. Functional imaging and modeling of the heart.

[CR25] Richardson WJ, Clarke SA, Quinn TA (2015). Physiological implications of myocardial scar structure. Compr Physiol.

[CR26] Sakai K, Watanabe K, Millard R (1985). Defining the mechanical border zone: a study in the pig heart. Am J Physiol.

[CR27] Schwinger RH, Böhm M, Koch A (1994). The failing human heart is unable to use the frank-starling mechanism. Circ Res.

[CR28] Shimkunas R, Makwana O, Spaulding K (2014). Myofilament dysfunction contributes to impaired myocardial contraction in the infarct border zone. Am J Physiol Heart Circ Physiol.

[CR29] Sutton MGSJ, Sharpe N (2000). Left ventricular remodeling after myocardial infarction: pathophysiology and therapy. Circulation.

[CR30] Tsao CW, Aday AW, Almarzooq ZI (2022). Heart disease and stroke statistics-2022 update: a report from the American Heart Association. Circulation.

[CR31] Tsuda T (2021). Clinical assessment of ventricular wall stress in understanding compensatory hypertrophic response and maladaptive ventricular remodeling. J Cardiovasc Dev Dis.

[CR32] Walker JC, Ratcliffe MB, Zhang P (2005). Mri-based finite-element analysis of left ventricular aneurysm. Am J Physiol Heart Circ Physiol.

[CR33] Weber KT, Clark WA, Janicki JS (1987). Physiologic versus pathologic hypertrophy and the pressure-overloaded myocardium. J Cardiovasc Pharmacol.

[CR34] Xie M, Burchfield JS, Hill JA (2013). Pathological ventricular remodeling: mechanisms: part 1 of 2. Circulation.

[CR35] Yao Y, Li A, Wang S (2022). Multifunctional elastomer cardiac patches for preventing left ventricle remodeling after myocardial infarction in vivo. Biomaterials.

[CR36] Zhuan X, Luo X, Gao H (2019). Coupled agent-based and hyperelastic modelling of the left ventricle post-myocardial infarction. Int J Numer Meth Biomed Eng.

